# Recent advances in understanding the ryanodine receptor calcium release channels and their role in calcium signalling

**DOI:** 10.12688/f1000research.16434.1

**Published:** 2018-11-27

**Authors:** Angela F. Dulhunty, Nicole A. Beard, Marco G. Casarotto

**Affiliations:** 1 Eccles Institute of Neuroscience, John Curtin School of Medical Research, 131 Garran Road, The Australian National University, Acton, ACT, 2601, Australia; 2 Centre for Research in Therapeutic Solutions, Faculty of Science and Technology, University of Canberra, Bruce, ACT, 2617, Australia; 3 Department of Cancer Biology and Therapeutics, John Curtin School of Medical Research, 131 Garran Road, The Australian National University, Acton, ACT, 2601, Australia

**Keywords:** Ryanodine Receptor, Skeletal and Cardiac Muscle, High Resolution Cryo-EM

## Abstract

The ryanodine receptor calcium release channel is central to cytoplasmic Ca ^2+^ signalling in skeletal muscle, the heart, and many other tissues, including the central nervous system, lymphocytes, stomach, kidney, adrenal glands, ovaries, testes, thymus, and lungs. The ion channel protein is massive (more than 2.2 MDa) and has a structure that has defied detailed determination until recent developments in cryo-electron microscopy revealed much of its structure at near-atomic resolution. The availability of this high-resolution structure has provided the most significant advances in understanding the function of the ion channel in the past 30 years. We can now visualise the molecular environment of individual amino acid residues that form binding sites for essential modulators of ion channel function and determine its role in Ca ^2+^ signalling. Importantly, the structure has revealed the structural environment of the many deletions and point mutations that disrupt Ca ^2+^ signalling in skeletal and cardiac myopathies and neuropathies. The implications are of vital importance to our understanding of the molecular basis of the ion channel’s function and for the design of therapies to counteract the effects of ryanodine receptor-associated disorders.

## Introduction

**Figure 1.**
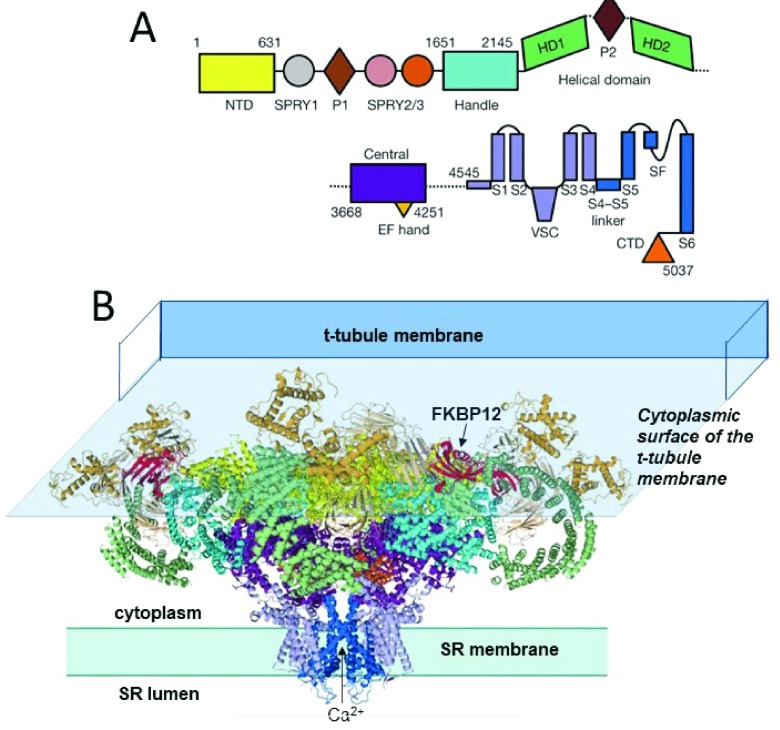
The near-atomic resolution structure of RyR1 ^10^. ( **A**) The linear sequence of RyR1 domains, starting at the N-terminal domain (NTD). ( **B**) “Side” view of RyR1 highlighting two of four protomers. Domains in ( **B**) are colour-coded in the same way as those in ( **A**). Modified from Figure 1 of Yan *et al*. ^10^. The 10 major domains in each subunit include the N-terminal domain, which harbours many RyR1 and RyR2 disease-causing mutations. They also include the SPRY1 domain that forms part of the binding site for FKPB12, the helical and central domains, which contain protein kinase A and Ca ^2+^/calmodulin protein kinase II (CaMKII) phosphorylation sites, the transmembrane domain containing the ion pore, and the C-terminal domain that forms part of the Ca ^2+^, ATP, and caffeine activation sites ^17^ ( Figure 2). FKBP12 binds in a cleft formed by the Handle, NTD, and SPRY1/3 domains. Suggested FKBP12 binding residues are located in the Handle domain (P1780, C1781, and S1687) ^10^ and in a hydrophobic cluster around D720 in the SPRY1 domain ^26^. The cytoplasmic surface of the transverse tubule (T-tubule) membrane is shown overlying the RyR to illustrate the way that the bulk of the RyR is sandwiched between the membranes on either side of the T-tubule/sarcoplasmic reticulum (SR) junction. RyR, ryanodine receptor.

Intracellular Ca ^2+^ signalling in many tissues depends on Ca ^2+^ ions cycling between the bulk of the cytoplasm and specialised intracellular Ca ^2+^ stores in endoplasmic reticulum (ER). Ca ^2+ ^is released from the stores through two classes of ion channel: the inositol 1,4,5-trisphosphate receptor (IP3R) and the ryanodine receptor (RyR) ^1^. Muscle contraction is almost entirely dependent on a massive outflow of Ca ^2+^ from the modified ER—sarcoplasmic reticulum (SR)—through RyR channels; relaxation then follows the return of Ca ^2+^ to the store by SERCA (sarcoplasmic endoplasmic reticulum calcium ATPase) pumps ^1^. The RyR opens in response to an action potential during excitation-contraction (EC) coupling. The action potential travels along the fibre surface and throughout the muscle fibre along transverse tubule extensions of the surface membrane, which forms multiple junctions with the SR membrane ^2^. The gap between the membranes is filled almost entirely by the cytoplasmic bulk of the RyR, which is embedded in the SR membrane ( Figure 1). In skeletal muscle, the action potential is detected by a surface membrane voltage sensor in the α _1S_ subunit of the dihydropyridine receptor (DHPR, also known as Ca _V1.1_) ^3, 4^. The signal from the action potential is transmitted to the RyR through a series of protein–protein interactions that are not yet fully defined but likely include the β _1a_ subunit of the DHPR and the STAC3 protein ^5, 6^. A simpler EC coupling system exists in the heart, where an action potential induces Ca ^2+^ entry through the cardiac DHPR (Ca _V1.2_) and the entering Ca ^2+^ ions activate the cardiac RyR2 in a Ca ^2+^-activated Ca ^2+^ release process ^1^. The massive RyR ion channel protein is composed of four monomers of more than 500 KDa. Its efficient operation depends on a myriad of factors that interact with its many domains. Such factors include associated proteins such as calmodulin, the FK506 binding proteins, and CLIC2, all of which bind to its large cytoplasmic domain. Binding to its tiny luminal domain are triadin, junctin, and the Ca ^2+^ binding protein calsequestrin ^5^. Other essential factors regulating the ion channel open probability and gating behaviour include post-translational modifications such as phosphorylation, oxidation, nitrosylation, nitration, and glutathiolation ^7, 8^. Acquired changes in channel function and genetically transmitted modifications can severely disrupt the ion channel’s function and detrimentally alter Ca ^2+^ signalling to lead to skeletal and cardiac myopathies that can be debilitating and often fatal. Despite the essential function of this ion channel protein, the molecular nature of its gating mechanisms and how they are disrupted in myopathies have remained elusive. Such insight required near-atomic resolution structures of the protein, which are now available with a resolution of not more than 6.1 Å following recent advances in cryo-electron microscopy (cryo-EM) ^9– 16^. Thus far, the more structurally stable RyR1 protein has been determined at the highest resolution of about 3.8 Å ^10, 13^ ( Figure 1). The highest resolution is achieved for the most stable parts of the protein, particularly the C-terminal portion encompassing the transmembrane/pore domains. The pore itself has been determined with highest resolution ^17^. Consequently, the high-resolution cryo-EM structure of the RyR protein is only partially resolved.

## The impact of high-resolution structures of the ryanodine receptor

**Figure 2.**
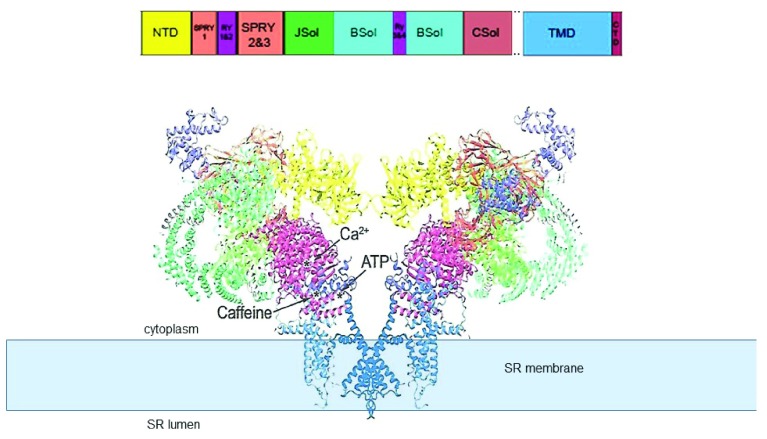
“Side view” of the open RyR1 channel structure ^9^. The structure (PDB code 5TAL) reveals the major domains of the protein and the location of Ca ^2+^, ATP, and caffeine binding sites identified by 13. Adapted from 17; the structural domain nomenclature is as given in 10. The transmembrane domain (TMD) containing the permeation pathway is shown embedded in the sarcoplasmic reticulum (SR) membrane, which is depicted as a solid pale blue rectangle. NTD, N-terminal domain; RyR, ryanodine receptor.

The enormous impact of the higher-resolution structures on our understanding of RyR function has been elegantly presented in full reviews by Meissner ^17^, Samsó ^18^, Zalk and Marks ^19^, and Santulli *et al*. ^20^. In particular, Meissner ^17^ and Samsó ^18^ provide excellent summaries of the different terminology applied to the various structural domains of the RyR, which have evolved in different ways as increasingly higher-resolution structures have been reported over the last 20 years. The various domains of RyR1 are shown in Figure 1 and Figure 2 and are based on Yan *et al*. ^10^ and Zalk *et al*. ^9^, respectively, and illustrate very different terminologies used by different authors. Different terminologies and residue numbers are summarised in Table 1 of 17. There are 10 significant domains in each RyR subunit: (1) the N-terminal domain, which is a hot spot in RyR1 and RyR2 for disease-causing mutations; (2) the SPRY1 domain that is part of the binding site for FKPB12 and, with SPRY3, forms a major part of the “clamp” region (older nomenclature) that closely opposes the T-tubule membrane and may interact indirectly with the DHPR α _1S_ subunit in EC coupling; (3) the Ry 1 and 2 or P2 domains; (4) the SPRY2 and 3 domain; (5) the junctional solenoid (Jsol or Handle), which also contributes to the FKBP binding pocket (see legend to Figure 1); (6, 7) bridging solenoid (Bsol encompassing helical domains 1 and 2) and containing the protein kinase A (PKA) and Ca ^2+^/calmodulin (CaM)-dependent protein kinase II (CaMKII) phosphorylation sites; (8) the core solenoid (Csol or central domain), which contains two EF hand Ca ^2+^ binding motifs; (9) the transmembrane domain (TMD) containing the Ca ^2+ ^pore with binding sites for ryanodine and the luminal domain of the RyR with triadin and junctin binding sites; and (10) the C terminal domain (CTD) that forms part of the Ca ^2+^, ATP, and caffeine activation site.

The Ca ^2+^, ATP, and caffeine binding sites have been identified by comparing differences between RyR1 with Ca ^2+^, but not ATP or caffeine, and RyR1 with ATP and caffeine bound, but without Ca ^2+^ ^13^. All three activators bind within a small region of the protein in which the CTD comes into close approximation with the Csol domain and TMD. Ca ^2+^ binds at the interface between the CTD and CSol. ATP binds at the junction between the CTD, the cytoplasmic extension of the S6 transmembrane helix, and the “thumb and forefinger” region adjacent to CSol. Caffeine binds between the CTD and cytoplasmic extension of the S6 helix. As predicted from many previous biochemical and physiological studies ^21– 24^, the ryanodine binding site is within the pore and adjacent to residue Q4933.

Of particular importance to muscle physiology are the substantial differences between the structures of the skeletal (RyR1) and cardiac (RyR2) isoforms, which explain many of the functional differences between the proteins and their role in Ca ^2+^ signalling in the two tissues ^17, 18, 25^. Also of major importance for basic muscle physiology/biophysics are the structural changes determining ion channel gating between the open and closed states. The recent high-resolution structures of RyRs locked into open or closed conformations, in combination with previous lower-resolution structural studies, allow refined models of the ion channel gating mechanisms to be developed ^13, 14, 16, 18, 20^. This has been no easy task, as the ion channel opening and closing require the coordination of more than 40 domains and the transmission of allosteric changes from both cytoplasmic domains and luminal domains to the channel gating mechanism. As stated by Samsó ^18^, “the RyR is the quintessential allosteric machine”. Detailed summaries of RyR1 and RyR2 domain structures are given in several recent publications ^13, 14, 16, 18, 20, 25^. In addition, defined locations of essential ATP, caffeine, and Ca ^2+^ binding sites in RyR1 are reported by des Georges *et al*. ^13^, and these observations provide a basis for predictions of the location of similar sites in the RyR2 protein ^27^. It became apparent that the channel can exist in a variety of conformations, not simply “open or closed”. Indeed, priming changes in the C-terminal activation module can occur without altering pore structure, and there may be a hierarchy of pore structure changes that depend on binding of Ca ^2+^, ATP, or caffeine that have not yet been determined but can produce incremental changes in channel open probability ^13^. It is worth noting that the structure of the open channel may continue to be further refined as optimal conditions, including the lipid content of preparative solutions, are developed for cryo-EM structural studies ^16^.

Mutations in RyRs can lead to severe genetic conditions, including malignant hyperthermia (MH) and central core disease (CCD) in skeletal muscle ^28^ and arrhythmia in cardiac muscle ^29^. Locating the mutated residues in the full-length channel structure has been difficult in the past but is now possible with the high-resolution cryo-EM RyR structures ^20^. Although such mutations can occur at almost any region in the protein, there are specific “hot spots”. MH occurs when RyR1 is activated by a volatile anaesthetic and leads to a rapid rise in core body temperature and organ failure unless treated ^30^. MH mutations generally occur in central (Bsol) and N-terminal clusters in regions that would impact on pore opening and lead to uncontrollable Ca ^2+^ release in the presence of anaesthetics ^20^. CCD is characterised histologically by cores of inactive tissue in the centre of muscle fibres that lead to progressive muscle weakness ^28^. RyR1 mutations causing CCD are clustered in the C-terminal Ca ^2+^ binding site at the interface between the CTD and CSol, where they can directly affect the activation mechanism ^20^. Mutations in the cardiac RyR2 that lead to arrhythmias are generally located in the pore, transmembrane, and central domain regions, which are directly involved in channel activation and gating ^13– 20^.

Importantly, the detailed three-dimensional (3D) locations of disease-causing mutations in RyR1 and RyR2 and determination of structural changes associated with mutations that lead to disrupted Ca ^2+^ signalling will help in understanding the molecular process involved in regulating the ion channel gating mechanisms. In addition, the structure of potential drug binding sites opens the possibility of rational design of therapeutics to correct defective RyR channel function and restore healthy Ca ^2+^ signalling ^20, 25, 31, 32^. As an aside, the high-resolution structural studies have provided evidence supporting early insightful hypotheses that the functional consequences of some of the disease-causing mutations are caused by “unzipping” of essential inter-domain interactions and thereby favour the open unstable conformation of the channel ^33– 35^.

It is worth noting that X-ray crystallography has been used to predict the structure of individual elements within the RyR. X-ray crystal structures of isolated RyR domains at resolutions of less than 2 Å have been obtained and have defined the structures of ligand binding sites and also revealed structural changes induced by ligand binding or caused by mutations ^33^. X-ray crystal structures have been docked into the 3D envelope of RyR calculated from cryo-EM data to help interpret the high-resolution structures reported for RyR1 ^9– 11^. This is further discussed in 17, 19, 36. The potential power of combining atomic resolution X-ray crystallography and nuclear magnetic resonance with cryo-EM to obtain high-resolution structures of large proteins of more than 500 KDa is discussed in detail in a review by Vénien-Bryan *et al*. ^36^.

**Figure 3.**
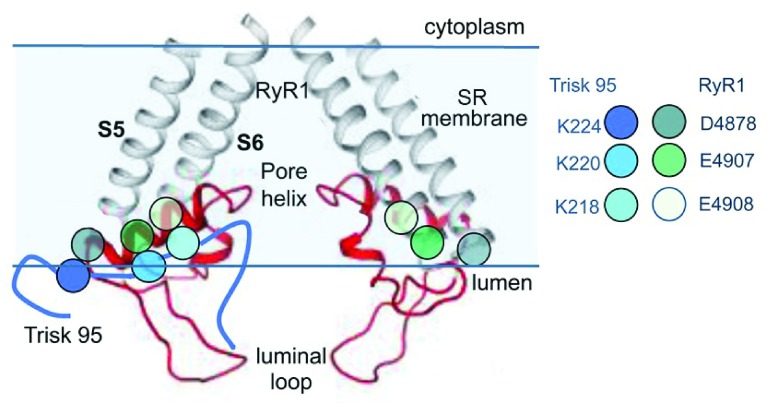
Proposed region for ionic interactions between skeletal triadin (Trisk 95) and RyR1. The near-atomic resolution structure of the pore-forming elements of RyR1 showing two diagonal protomers (Extended Data Figure 8 in ^10^). The grey transmembrane S5 and S6 helices and red pore helix, S5–pore helix linker, and SF linker between the pore helix and S6 helix are shown. Mutagenesis studies suggest that residues D4878, E4907, and E4908 in the outer regions of the pore helix are associated with K218, K220, and K224 in Trisk 95 ^39, 40^. The approximate positions of E4907 and E4908 are indicated by the black arrowhead, and the arrow indicates the predicted binding site for Trisk 95. RyR, ryanodine receptor; SR, sarcoplasmic reticulum. Reprinted by permission from Springer Nature: Pflügers Archiv European Journal of Physiology, Three residues in the luminal domain of triadin impact on Trisk 95 activation of skeletal muscle ryanodine receptors, E. Wium, A. F. Dulhunty, N. A. Beard, © 2016.

The recent high-resolution cryo-EM structures have been pivotal in revealing the truly minute nature of the luminal domain of the RyR protein. Residues that comprise the “luminal” domain can be defined as those that extend from the SR membrane into the luminal compartment ( Figure 1 and Figure 2). The parts of the protein extending beyond the membrane likely consist only of short luminal loops linking the transmembrane helices 5 and 6 and the pore helix and thus constitute less than 1.5% of the protein mass ^5^. However, as the hydrophilic luminal environment extends into the highly charged domains of the channel pore, the actual amount of the protein exposed to the luminal solution is greater than the luminal loops that extend beyond the generalised membrane boundary. Nevertheless, it is remarkable that the luminal domain is relatively very small given the importance of luminal conditions in determining the open probability of RyR1 and RyR2 channel *in vivo* and *in vitro*. The open probability of the RyR channel is very sensitive to changes in luminal [Ca ^2+^], and changes in the RyR’s sensitivity to luminal Ca ^2+^ underlie the defects in Ca ^2+^ signalling that produce skeletal and cardiac myopathies ^37, 38^. In addition, the luminal domains provide the RyR binding sites for the associated regulatory proteins triadin and junctin ( Figure 3) ^39, 40^. These proteins anchor the RyR to the luminal Ca ^2+^ binding protein calsequestrin, and all three proteins impact on RyR1 and RyR2 activity. Mutations in any of the three luminal proteins can alter the functional interactions between the proteins and the luminal domain of the RyR. Such altered interactions lead to significant changes in RyR function and Ca ^2+^ signalling and produce significant cardiac and skeletal myopathies ^5^.

## Concluding comments

The high-resolution cryo-EM revolution over the past four years has had a major impact on the interpretation of RyR physiology in terms of the specific structural changes within the RyR protein. The structure of the protein in the open and closed state reveals conformational changes in the pore that allow Ca ^2+^ flow from the SR and significant associated changes in the structure of remote cytoplasmic domains of the protein. The nature of structural re-organisation resulting from post-translational modifications and mutations can now be predicted and the predictions tested and refined in future cryo-EM studies of RyRs subjected to specific modifications or mutations. It is likely that some of the current structures, particularly those of the open conformations, will be further refined as yet higher-resolution structures become available and improved isolation conditions are developed.

It is also likely that the next major structural development will be the determination of the structure of the RyR in the intact adult mammalian muscle fibre, ideally in the environment of the junctional gap that separates the surface (T-tubule) membrane and the SR. This will reveal the RyR1 structure when it is associated with other proteins that are essential for EC coupling such as the DHPR α and β subunits, STAC3, and junctophilin. For those in the field, it is a continual frustration that we know these proteins are essential for EC coupling and we can construct models for their interactions but that we will never fully understand skeletal EC coupling until we have determined their 3D associations within the intact cell. This next step awaits the development of high-resolution tomography techniques, possibly cryo-EM tomography ^18^. Indeed, high-resolution tomography may also reveal the way in which RyR channels associate with each other in the SR membrane to allow “coupled gating” between neighbours, a phenomenon that is seen in lipid bilayers ^41– 44^ and that is thought to be critical to RyR function in muscle fibres ^20, 45^.
